# Supplementary material for: Causal Relationship between Aging and Anorexia Nervosa: A White-Matter-Microstructure-Mediated Mendelian Randomization Analysis
Source: Biomedicines. 2024 Aug 16;12(8):1874. doi: 10.3390/biomedicines12081874 (PMC11351342; doi:10.3390/biomedicines12081874)
Supplement: Supplementary file 1 [file biomedicines-12-01874-s001.zip › Supplementary Figure/Figure S4/Singe SNP plot of MR analysis for TL to ubm-b-1618.pdf]

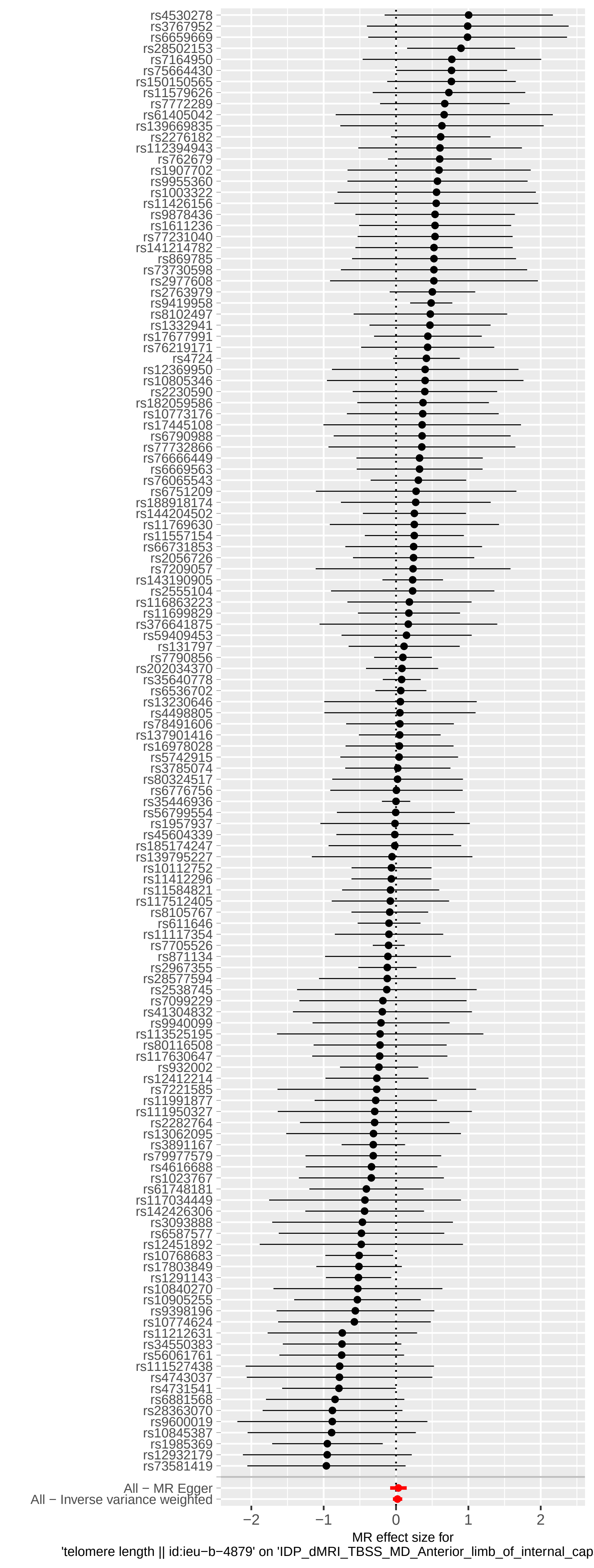
rs4530278
rs3767952
rs6659669
rs28502153
rs7164950
rs75664430
rs150150565
rs11579626
rs7772289
rs61405042
rs139669835
rs2276182
rs112394943
rs762679
rs1907702
rs9955360
rs1003322
rs11426156
rs9878436
rs1611236
rs77231040
rs141214782
rs869785
rs73730598
rs2977608
rs2763979
rs9419958
rs8102497
rs1332941
rs17677991
rs76219171
rs4724
rs12369950
rs10805346
rs2230590
rs182059586
rs10773176
rs17445108
rs6790988
rs77732866
rs76666449
rs6669563
rs76065543
rs6751209
rs188918174
rs144204502
rs11769630
rs11557154
rs66731853
rs2056726
rs7209057
rs143190905
rs2555104
rs116863223
rs11699829
rs376641875
rs59409453
rs131797
rs7790856
rs202034370
rs35640778
rs6536702
rs13230646
rs4498805
rs78491606
rs137901416
rs16978028
rs5742915
rs3785074
rs80324517
rs6776756
rs35446936
rs56799554
rs1957937
rs45604339
rs185174247
rs139795227
rs10112752
rs11412296
rs11584821
rs117512405
rs8105767
rs611646
rs11117354
rs7705526
rs871134
rs2967355
rs28577594
rs2538745
rs7099229
rs41304832
rs9940099
rs113525195
rs80116508
rs117630647
rs932002
rs12412214
rs7221585
rs11991877
rs111950327
rs2282764
rs13062095
rs3891167
rs79977579
rs4616688
rs1023767
rs61748181
rs117034449
rs142426306
rs3093888
rs6587577
rs12451892
rs10768683
rs17803849
rs1291143
rs10840270
rs10905255
rs9398196
rs10774624
rs11212631
rs34550383
rs56061761
rs111527438
rs4743037
rs4731541
rs6881568
rs28363070
rs9600019
rs10845387
rs1985369
rs12932179
rs73581419
All − MR Egger
All − Inverse variance weighted
−2
−1
0
1
2
MR effect size for
'telomere length || id:ieu−b−4879' on 'IDP_dMRI_TBSS_MD_Anterior_limb_of_internal_cap
